# Genomic Characterization of β-Glucuronidase–Positive *Escherichia coli* O157:H7 Producing Stx2a 

**DOI:** 10.3201/eid2412.180404

**Published:** 2018-12

**Authors:** Yoshitoshi Ogura, Kazuko Seto, Yo Morimoto, Keiji Nakamura, Mitsuhiko P. Sato, Yasuhiro Gotoh, Takehiko Itoh, Atsushi Toyoda, Makoto Ohnishi, Tetsuya Hayashi

**Affiliations:** Kyushu University, Fukuoka, Japan (Y. Ogura, K. Nakamura, M.P. Sato, Y. Gotoh, T. Hayashi);; Osaka Institute of Public Health, Osaka, Japan (K. Seto);; Hokkaido Institute of Public Health, Hokkaido, Japan (Y. Morimoto);; Tokyo Institute of Technology, Tokyo, Japan (T. Itoh);; National Institute of Genetics, Shizuoka, Japan (A. Toyoda);; National Institute of Infectious Diseases, Tokyo (M. Ohnishi)

**Keywords:** Escherichia coli, GP STEC O157:H7, genome, Shiga toxin, Stx2a, bacteria, β-glucuronidase, STEC, enteric infections

## Abstract

Among Shiga toxin (Stx)–producing *Escherichia coli* (STEC) O157:H7 strains, those producing Stx2a cause more severe diseases. Atypical STEC O157:H7 strains showing a β-glucuronidase–positive phenotype (GP STEC O157:H7) have rarely been isolated from humans, mostly from persons with asymptomatic or mild infections; Stx2a-producing strains have not been reported. We isolated, from a patient with bloody diarrhea, a GP STEC O157:H7 strain (PV15-279) that produces Stx2a in addition to Stx1a and Stx2c. Genomic comparison with other STEC O157 strains revealed that PV15-279 recently emerged from the *stx1a/stx2c*-positive GP STEC O157:H7 clone circulating in Japan. Major virulence genes are shared between typical (β-glucuronidase–negative) and GP STEC O157:H7 strains, and the Stx2-producing ability of PV15-279 is comparable to that of typical STEC O157:H7 strains; therefore, PV15-279 presents a virulence potential similar to that of typical STEC O157:H7. This study reveals the importance of GP O157:H7 as a source of highly pathogenic STEC clones.

Shiga toxin (Stx)–producing *Escherichia coli* (STEC) with the serotype O157:H7 is characterized by the possession of *stx* gene(s), the locus of enterocyte effacement (LEE) –encoded type 3 secretion system (T3SS), and a large virulence plasmid (pO157) that encodes enterohemolysin and other virulence factors, such as catalase-peroxidase KatP, a type II secretion system, and the protease EspP (*1*). Non–sorbitol-fermenting (NSF) and β-glucuronidase–negative (GN) STEC O157:H7 (hereafter referred to as typical STEC O157:H7), the major clone among currently circulating STEC O157:H7 strains, frequently causes large outbreaks of severe enteric infections, including diarrhea, hemorrhagic colitis, and hemolytic uremic syndrome. Stxs are classified as Stx1 or Stx2; Stx1 currently includes 3 subtypes (*stx1a*, *stx1c*, and *stx1d*) and Stx2 includes 7 subtypes (*stx2a* to *stx2g*) (*2*). The *stx* genes are encoded by lambda-like phages and have been acquired by STEC strains through phage transduction (*3*). Typical STEC O157:H7 produces Stx1a, Stx2a, and Stx2c, either alone or in combination. Stx2a-producing strains cause more severe infections than do Stx1a-producing strains (*4*). In addition, the levels of Stx2 production among the STEC O157:H7 strains carrying *stx2c*, but not *stx2a*, are typically very low (*5**–**7*).

According to the stepwise evolution model (*8**,**9*), STEC O157:H7 evolved from ancestral enteropathogenic *E. coli* (EPEC) O55:H7 (sorbitol-fermenting [SF] and β-glucuronidase–positive [GP]; clonal complex [CC] A1) through sequential acquisitions of virulence factors and phenotypic traits along with a serotype change (Figure 1). Two phenotypic variants of STEC O157 are known: SF/GP STEC O157:H– (nonmotile) (CC A4), known as the German clone (SF STEC O157:H–), and NSF/GP STEC O157:H7 (CC A5) (GP STEC O157:H7). Both variants are postulated to have emerged from a hypothetical intermediate, CC A3; typical STEC O157:H7 (CC A6) has further emerged from CC A5.

**Figure 1:**
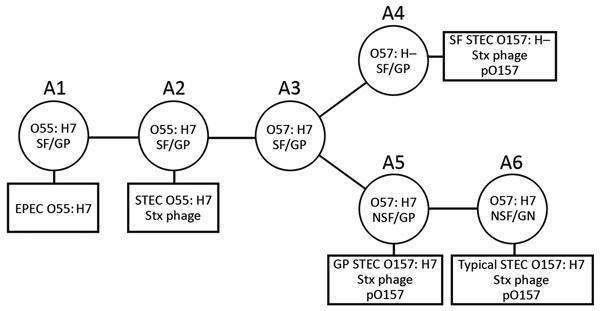
Schematic illustrating model of the stepwise evolution of STEC O157. The proposed stepwise evolution model of STEC O157 was schematically illustrated according to previous reports (*8**,**9*). Clonal complexes (CCs) A1 to A6 are indicated, along with phenotypic changes, antigen shifts, and acquisitions of Stx phages and pO157. Squares indicate contemporary circulating STEC O157 clones. EPEC, enteropathogenic *E. coli*; GP, β-glucuronidase–positive; NSF, non–sorbitol-fermenting; SF, sorbitol-fermenting; STEC, Stx–producing *E. coli*.

SF STEC O157:H– strains are usually *stx2a* positive (*10*); like typical STEC O157:H7, they have caused many outbreaks and sporadic cases of hemolytic uremic syndrome in Germany and other countries in Europe. Therefore, this clone is generally thought to be highly pathogenic (*11*–*13*). In contrast, although GP STEC O157:H7 strains have been reported to carry both *stx1* and *stx2* or only *stx2* (*14*–*16*), strains producing the Stx2a subtype have not been described. Although GP STEC O157:H7 was first isolated from a patient with bloody diarrhea in 1994 (*14*), this variant has rarely been isolated from humans. Moreover, human isolates obtained to date have generally been isolated from patients with asymptomatic or mild infections (*16*). The genomic information available for GP STEC O157:H7 is also limited to strain G5101 (*9*,*17*) and 4 strains in public databases. Thus, the virulence potential of GP STEC O157:H7 remains to be fully elucidated.

In this study, we isolated a GP STEC O157:H7 strain that produces Stx2a, in addition to Stx1a and Stx2c, from a patient with bloody diarrhea in Japan and determined its complete genome sequence. To reveal the genomic features of the Stx2a-producing strain, we determined the draft genome sequences of an additional 13 GP STEC O157:H7 isolates, which are all Stx2a negative but Stx1a/Stx2c (or Stx2c) positive, and performed fine phylogenetic and genomic comparisons of these GP STEC O157:H7 strains with typical STEC O157:H7 and SF STEC O157:H– strains. We also analyzed the Stx production levels of GP STEC O157:H7 strains.

## Methods

### Bacterial Strains

The strains used in this study are listed in Technical Appendix Table 1. The GP STEC O157:H7 strain PV15-279 was isolated from an adult patient in Japan who was hospitalized in 2015 with severe symptoms, including bloody diarrhea. The other 13 GP STEC O157:H7 strains sequenced in this study were isolated in Japan during 1988–2013 from humans with or without symptoms. We obtained genome sequence information for 3 *E. coli* O55:H7 (*18**–**20*), 2 SF STEC O157:H–, 14 typical STEC O157:H7 (*21**–**26*), and 2 GP STEC O157:H7 strains (*17*) from public databases.

### Subtyping of *stx* Genes

We performed in silico subtyping of *stx1* and *stx2* in all strains analyzed in this study. We used blastn (https://blast.ncbi.nlm.nih.gov/Blast; >99% identity and >99% coverage) for comparisons with previously reported reference sequences (*2*).

### Genome Sequencing, Assembly, and Annotation

We determined the complete genome sequence of PV15-279 using PacBio reads obtained with an RS II system (PacBio, Menlo Park, CA, USA). We assembled the reads with Canu version 1.5 (*27*) and circularized them using Circlator (*28*). We obtained Illumina paired-end reads (300 bp × 2) with a MiSeq sequencer (Illumina, San Diego, CA, USA) and mapped them to the assembly using the Burrows-Wheeler aligner (*29*) for sequence-error correction with Pilon (*30*). We completed further corrections to the sequences corresponding to Stx phage regions using MiSeq reads obtained by sequencing long-range PCR products covering each Stx phage. We performed annotations with the DDBJ Fast Annotation and Submission Tool (*31*), followed by manual curation using the IMC-GE software (In Silico Biology, Kanagawa, Japan).

We obtained the draft genome sequences of 13 GP STEC O157:H7 strains by assembling Illumina paired-end reads using Platanus (*32*). The Illumina reads for strain LB473017, for which the only read data available were from public databases, were also assembled with Platanus.

The genome sequences of PV15-279 and the 13 GP STEC O157:H7 strains have been deposited in DDBJ/EMBL/GenBank under the Bioproject accession numbers PRJDB6584 and PRJDB6498, respectively. The accession numbers of each sample, including the reference data, are listed in Technical Appendix Table 1.

### Single-Nucleotide Polymorphism Detection and Phylogenetic Analysis

We performed single-nucleotide polymorphism (SNP) detection and phylogenetic analyses as described previously (*33*). The genome sequences to be examined were aligned with the phage- and insertion sequence (IS)–masked chromosome sequence of the STEC O157:H7 strain Sakai (*22*) using MUMmer (*34*) to identify conserved regions (cutoff thresholds >98% sequence identity and >1,000-bp alignment length) in each strain and the SNP sites located therein. We then determined the core genome sequence that was conserved in all strains examined. Only SNPs located in the core genome were subjected to further analysis. After reconstructing the genome sequences of each strain using the SNPs and removing recombinogenic SNP sites using Gubbins (*35*), we constructed a maximum-likelihood phylogenetic tree using RAxML (*36*) with the general time-reversible plus gamma model of nucleotide substitution and 500 bootstrap replicates. The tree was displayed and annotated using iTOL (*37*).

### Repertoire Analysis of Genes Encoding T3SS Effectors and Plasmid-Encoded Virulence Factors

We analyzed the conservation of genes encoding T3SS effectors and plasmid-encoded virulence factors with blastn (>90% identity and >30% coverage). All intact effector genes and plasmid virulence genes identified in strains Sakai and PV15-279 were clustered using CD-HIT (*38*) at >90% identity and >30% alignment coverage of the longer sequences, and representative sequences of each cluster were used to create a database for blastn analysis.

### Stx Phage Sequencing

We determined the complete sequences of the Stx1a and Stx2c phages from strain 980938 (from an asymptomatic carrier) and the Stx2c phage from strain 981447 (from a patient with bloody diarrhea) as described previously (*39*). We constructed fosmid libraries of the 2 strains using a CopyControl fosmid library production kit (Epicenter Biotechnologies, Madison, WI, USA). We screened the *stx1-* or *stx2*-containing clones using PCR and sequenced them by the shotgun sequencing strategy using an ABI3730 sequencer (Applied Biosystems, Foster City, CA, USA).

### Stx Production Assay

We inoculated bacterial cells into 40 mL of CAYE broth (Merck, Darmstadt, Germany) and grew them to mid-log phase at 37°C with shaking. We then added mitomycin C (MMC) to the culture at a final concentration of 500 ng/mL. After MMC addition, we collected 100 μL of the culture every hour and immediately subjected it to sonication using a Bioruptor (Cosmo Bio, Tokyo, Japan). We obtained the soluble fractions of each cell lysate via centrifugation at 14,000 × *g* for 10 min at 4°C. We determined Stx1 and Stx2 concentrations in each cell lysate using a previously described sandwich ELISA (*40*). We captured Stx using RIDASCREEN Verotoxin microtiter plates (R-Biopharm, Darmstadt, Germany) coated with capture antibodies that recognize both Stx1 and Stx2. We conjugated monoclonal antibodies against Stx1 and Stx2 (LSBio, Seattle, WA, USA) with horseradish peroxidase using the Peroxidase Labeling Kit–NH_2_ (Dojindo, Kumamoto, Japan) and employed them as detection antibodies. We used reagents supplied in the RIDASCREEN Verotoxin kit for detection. Finally, we measured the absorbance at 450 nm (A_450_) using Tecan Infinite 200 PRO (Tecan, Männedorf, Switzerland).

## Results

### Isolation and Genome Sequencing of GP O157:H7 Strains

In PV15-279, we detected *stx2a* in addition to *stx1a* and *stx2c* (Technical Appendix Table 1). Determination of the complete genome sequence of PV15-279 revealed that the genome consisted of a 5,598,151-bp chromosome and a 94,391-bp plasmid. The draft genome sequences of 13 additional GP STEC O157:H7 strains were also determined for comparison (Technical Appendix Table 1). These strains were all *stx1a*/*stx2c*-positive and *stx2a*-negative. The *uidA* gene, which encodes β-glucuronidase, was intact in all the GP STEC O157:H7 strains sequenced in this study, as well as in 2 GP STEC O157:H7 strains whose draft genome sequences were publicly available (Technical Appendix Table 1). In typical STEC O157:H7, *uidA* has been inactivated by a frameshift mutation (*41*).

### Comparisons of General Genomic Features and Mobile Genetic Elements

**Figure 2:**
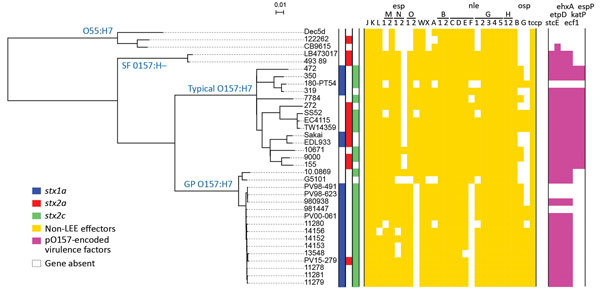
Whole-genome sequence-based phylogenetic analysis and repertoires of T3SS effectors and plasmid-encoding virulence factors from the study of Stx–producing *E. coli* O157:H7. The genome sequences of all the strains used in this study were aligned with the complete chromosome sequence of Sakai, and the single-nucleotide polymorphisms located in the 4,074,209-bp backbone sequence that were conserved in all the test strains were identified. After removing the recombinogenic single-nucleotide polymorphisms sites, we performed a concatenated alignment of 5,803 informative sites to generate the maximum-likelihood phylogeny. The conservation of T3SS effectors and plasmid-encoding virulence factors is shown in the tree. Colored boxes indicate the presence, and open boxes the absence, of each gene. GP, β-glucuronidase–positive; LEE, locus of enterocyte effacement; SF, sorbitol-fermenting. Scale bars indicate nucleotide substitutions per site.

Although the chromosome of PV15-279 was ≈100 kb larger than that of the typical GP STEC O157:H7 strain Sakai (Table and Technical Appendix Figure 1), the chromosome backbone was highly conserved; 97.2% of the coding sequences (CDSs) identified in Sakai were conserved in PV15-279. Among the various types of mobile genetic elements (MGEs), integrative elements (defined as elements that contain an integrase gene but no other MGE-related genes) were well conserved and showed only minor variations. Among the 5 integrative elements identified in Sakai, 1 small element (SpLE2) was missing in PV15-279, and multiple small structural variations were observed in 3 elements (Technical Appendix Table 2, Figure 2, panel B). In contrast, the prophage contents exhibited notable differences. PV15-279 contained 22 prophages, whereas Sakai contained 18 prophages. This difference is attributable mainly to the difference in chromosome sizes between the 2 strains. Although most of the integration sites used by Sakai prophages (13/16) are also used by PV15-279 prophages (Technical Appendix Table 2, Figure 2, panel A), in most instances prophages that had integrated into the same site showed notable variations between the 2 strains, suggesting the existence of different evolutionary histories. Furthermore, PV15-279 carried as many as 4 Stx phages: 1 Stx1a phage, 2 Stx2a phages, and 1 Stx2c phage (Technical Appendix Figure 1). In contrast, Sakai carries 1 Stx1a phage and 1 Stx2a phage (see subsequent sections for a comparison of these phages with Stx phages from various STEC O157:H7 strains). Surprisingly, 1 of the 2 Stx2a phages (PV15p10) was integrated together with 3 prophages at the *ydfJ* locus in PV15-279 in tandem (Technical Appendix Table 2, Figure 1, Figure 2, panel A).

**Table:** General genomic features of the β-glucuronidase–positive STEC O157:H7 strain PV15-279 from Japan and the typical STEC O157:H7 strain Sakai*

Feature	PV15-279	Sakai
Chromosome		
Length, bp	5,598,152	5,498,578
CDSs (pseudogenes)	5,295 (107)	5,202 (122)
rRNA operons	7	7
tRNAs	108	104
Prophages	22	18
Integrative elements	5	6
IS elements	80	65
Plasmid pO157		
Length, bp	94,391	92,722
CDSs (pseudogenes)	95 (0)	90 (8)
IS elements	11	8
Plasmid pOSAK1		
Length, bp	NA	3,306
CDSs (pseudogenes)	NA	3 (0)
Total genome size, kb	5,692,969	5,591,300

*CDS, coding sequence; IS, insertion sequence; NA, not available; STEC, Shiga toxin–producing *Escherichia coli*.

The repertoires and total copy numbers of IS elements also exhibited notable differences (Technical Appendix Table 3). Although 24 types of IS elements were identified in Sakai and 18 types were identified in PV15-279, only 14 were shared by the 2 strains. A greater number of total copies of IS elements was detected in PV15-279 (91, compared with 73 in Sakai), primarily because of the proliferation of IS*1203* (also annotated as IS*629*) in PV15-279. The virulence plasmid pO157 also showed notable differences, as described in the next section.

### Phylogenetic Characterization of GP STEC O157:H7 Strains

To determine the precise phylogenetic position of PV15-279 in STEC O157, we performed high-resolution phylogenetic analysis using the genome sequences of various typical and atypical STEC O157 strains (Figure 2). The results clearly indicate that PV15-279 formed a distinct cluster with all other GP STEC O157:H7 strains, including 2 US isolates, and is a strain that recently emerged from the *stx1a/stx2c*-positive clone circulating in Japan. The phylogenetic relationship of this cluster with other STEC O157 lineages was concordant with the stepwise evolution model (Figure 1).

In our phylogenetic tree, the GP STEC O157:H7 strains were relatively clonal compared with the typical STEC O157:H7 strains, although these typical STEC O157:H7 strains were selected to represent their currently known phylogenetic diversity (*26*) (Figure 2). Despite the close phylogenetic relationship between the GP STEC O157:H7 strains, only PV15-279 was *stx2a-*positive, indicating that PV15-279 acquired *stx2a* very recently. No *stx* genes were detected in the publicly available sequence data for the GP STEC O157:H7 strain G5101, although this strain was previously reported to contain *stx1* and *stx2* (*14*).

### Distribution of T3SS Effectors in the GP O157:H7 Lineage

In addition to 5 LEE-encoded T3SS effectors, 18 families of effectors are encoded at non-LEE genomic loci (non-LEE effectors) in Sakai (*42*). In PV15-279, we identified most of the effector families found in Sakai (Technical Appendix Table 4). The major differences were the absence of *nleF* in PV15-279 and the presence of *ospB* and *ospG* in PV15-279, both of which are absent in Sakai. After subgrouping the 4 effector families (*espM, espN, espO*, and *nleG*) into 2–5 subgroups based on their sequence diversity, we analyzed the effector repertoires of the STEC O157 isolates used in the phylogenetic analysis (Figure 2). This analysis revealed that the effectors identified in PV15-279 were mostly conserved in other GP STEC O157:H7 strains, although some variations were detected.

### Virulence Plasmid pO157 of GP O157:H7 Lineage 

The pO157 plasmid of PV15-279 was nearly identical to the plasmid from the GP STEC O157:H7 strain G5101 (*43*), with several small variations that were apparently generated by mechanisms involving IS (Technical Appendix Figure 3). The plasmid-encoded virulence genes identified in PV15-279 were well conserved in other GP STEC O157:H7 strains, except for 3 strains from which pO157 has apparently been deleted (Figure 2). It is unknown whether this deletion occurred before or after strain isolation.

As previously reported (*43*), the pO157 plasmids from GP STEC O157:H7 showed high similarity to those from typical STEC O157:H7 and SF STEC O157:H–. Thus, a pO157-like plasmid was likely acquired by the common ancestor of the 3 STEC O157 lineages. A notable difference between the pO157 plasmids of the 3 STEC O157 lineages was the distribution of *katP* and *espP*. These genes were detected only in typical STEC O157:H7 strains, suggesting that these genes may have been specifically acquired by the typical STEC O157:H7 lineage. Although the roles of *katP* and *espP* in STEC virulence in humans have not yet been elucidated, at least the SF STEC O157:H– strain causes severe infections even without these genes.

### Stx Phages of GP STEC O157:H7 

**Figure 3:**
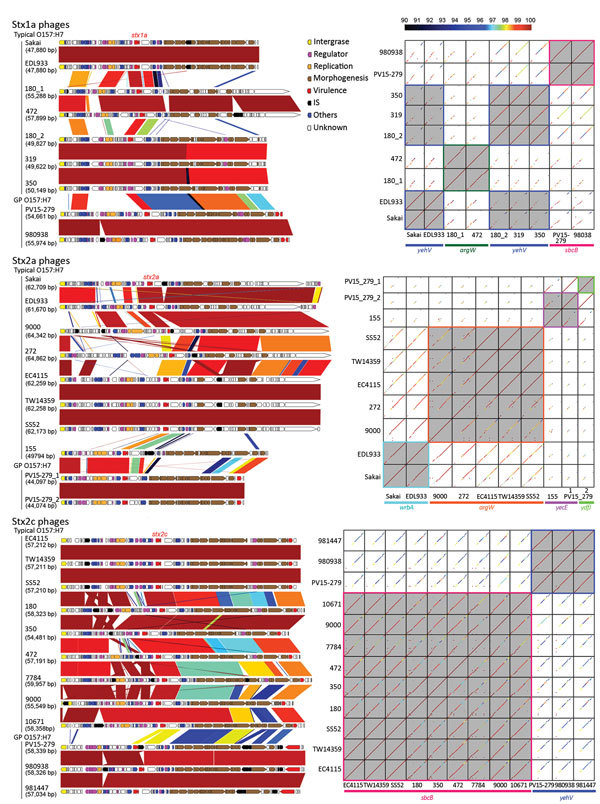
Genome comparisons of Stx phages from the study of Stx-producing *E. coli* O157:H7. The results of the comparison of the genome structure (left) and dot-plot sequence comparisons (right) of the Stx phages are shown. Sequence identities are indicated by different colors. In the dot-plot matrices, phages integrated in the same integration sites are highlighted by gray shading and colored frames. GP, β-glucuronidase–positive; IS, insertion sequence.

As shown in Figure 3, we performed fine genomic comparisons of the Stx phages from PV15-279 with the Stx1a, Stx2a, and Stx2c phages from other STEC O157:H7 strains used in the phylogenetic analysis shown in Figure 2 (phages were included only when complete sequences were available). The Stx1a and Stx2c phages of the GP STEC O157:H7 strain 980938 and the Stx2c phage of the GP STEC O157:H7 strain 981447 were sequenced individually and included in the analysis.

Stx1a phages were integrated in *sbcB* in the 2 GP STEC O157:H7 strains but were integrated in *yehV* or *argW* in typical STEC O157:H7 strains. According to the dot plot analysis, the 2 Stx1a phages from GP STEC O157:H7 were nearly identical (Figure 3). The phages from typical STEC O157:H7 strains were classified into 3 groups based on sequence similarity. The Stx1a phages from GP STEC O157:H7 showed various levels of sequence similarity to each group. The highest similarity was observed for the 3 phages integrated in *yehV*, but a clear difference was observed in the early region.

The 2 Stx2a phages from PV15-279 that integrated in *yecE* and *ydfJ* were nearly identical (only 2 SNPs), indicating that these phages were recently duplicated in this strain. With 1 exception (the phage from strain 155), the Stx2a phages from the typical STEC O157:H7 strains analyzed in this study were integrated in either *wrbA* or *argW*, and all shared a similar genomic structure, although considerable variations were observed in the early region, as reported previously (*7*). The Stx2a phages from PV15-279 exhibited a genetic structure similar to that of strain 155, which was also integrated in *yecE*. However, those phages showed high sequence similarity only in limited regions.

The Stx2c phages from GP STEC O157:H7 strains (all integrated in *yehV*) were nearly identical, showing only minor variations, such as IS integration. The Stx2c phages from typical STEC O157:H7 strains, which were all integrated in *sbcB*, also exhibited very similar genomic structures and sequences. Although notable sequence similarity was observed between the Stx2c phages from GP STEC O157:H7 and typical STEC O157:H7, the early regions were very different.

### Stx Production by GP STEC O157:H7 Strains

**Figure 4:**
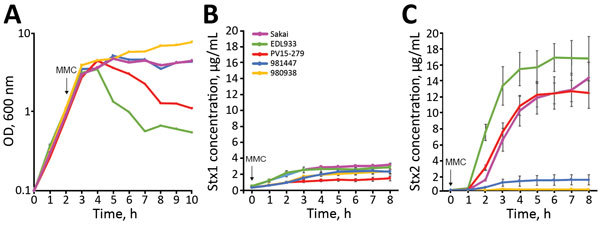
Lysis curves and levels of Stx produced by STEC O157:H7 strains after MMC treatment in study of Stx-producing *E. coli*. The lysis curves (A) and levels of Stx1 (B) and Stx2 (C) production by 5 STEC O157:H7 strains after the MMC treatment are shown. After the addition of MMC, the OD_600_ of each strain was measured every hour for 8 hours, and 100 μL of the culture was collected at each time point. Cell lysates were prepared, and the Stx1 and Stx2 concentrations in the soluble fractions were analyzed using a sandwich ELISA. All experiments were performed 3 times, and the averages and SEs of the Stx1 and Stx2 concentrations in each sample are plotted. Strains Sakai and EDL933 belong to typical STEC O157:H7, and the other 3 strains belong to GP STEC O157:H7. GP, β-glucuronidase–positive; MMC, mitomycin C; OD, optical density; STEC, Stx-producing *E. coli*.

Stx1 production is partially dependent on phage induction, whereas Stx2 production is strongly dependent on phage induction (*44*–*46*). We compared Stx production levels between GP STEC O157:H7 and typical STEC O157:H7 using MMC as a phage induction agent; we analyzed Stx1 and Stx2 production by representative strains of the 2 lineages (PV15-279, 981447, and 980938 for GP STEC O157:H7; Sakai and EDL933 for typical STEC O157:H7). MMC exhibited different levels of effectiveness in phage induction in each strain, and clear cell lysis (a clear reduction in OD_600_ values) was observed only in EDL933 and PV15-279 (Figure 4, panel A).

Consistent with a previous report (*47*), the Stx1 concentration was not notably elevated by phage induction in any of the strains, and no clear difference was observed between the GP STEC O157:H7 and typical STEC O157:H7 strains (Figure 4, panel B). In contrast, levels of Stx2 production were highly variable, as previously shown for typical STEC O157:H7 strains (*7*). Although Stx2 production was poorly induced in 2 GP STEC O157:H7 strains carrying *stx2c* alone, it was strongly induced in the *stx2a*-positive PV15-279 and typical STEC O157:H7 strains (Figure 4, panel C). Therefore, s*tx2a* was strongly induced in PV15-279, as in typical STEC O157:H7, but *stx2c* was poorly induced in PV15-279, as in the other GP STEC O157:H7 strains. The level of Stx2 production by PV15-279 was comparable to that of typical STEC O157:H7 strains and similar to that of the Sakai strain.

## Discussion

In this study, we isolated a GP STEC O157:H7 strain (PV15-279) that produces Stx2a in addition to Stx1a and Stx2c. The whole-genome sequence-based phylogenetic analysis, which included additional GP STEC O157:H7 strains and representative strains belonging to other *E. coli* O55/O157 lineages, revealed that PV15-279 recently emerged by the acquisition of Stx2a phage from the *stx1a/stx2c*-positive GP STEC O157:H7 clone circulating in Japan (Figure 2). Most of the major virulence genes identified in typical STEC O157:H7, such as T3SS effector genes and plasmid-encoded virulence genes, were well conserved in PV15-279 and other GP STEC O157:H7 strains, although some variations were detected (Figure 2). Moreover, the ability of PV15-279 to produce Stx2a was comparable to that of typical STEC O157:H7 (Figure 4). These findings suggest that PV15-279 presents a virulence potential similar to that of typical STEC O157:H7. In fact, PV15-279 was isolated from a patient who had severe enteric symptoms, including bloody diarrhea. However, further investigations are necessary to determine how the variations in the virulence gene repertoire detected in the comparison with other STEC O157 lineages affect the potential virulence of GP STEC O157:H7. The repertoires of the minor virulence genes of GP STEC O157:H7 must also be investigated.

We also obtained several noteworthy findings concerning the evolution of GP O157:H7 in this study. For example, fine genomic comparisons of Stx phages revealed that Stx phages differ notably between GP STEC O157:H7 and typical STEC O157:H7 (Figure 3). Although many of these variations may have been generated through extensive recombination with various resident or incoming phages (*48*), the acquisition of the Stx2a phage by the GP STEC O157:H7 strain PV15-279 was apparently an independent genetic event. The clear differences in the genetic structure, sequence, and integration sites of Stx2c phages between GP and typical STEC O157:H7 may suggest that Stx2c phages were also acquired independently by the 2 lineages after they separated. In contrast, the similar genetic structures and sequences of the pO157 plasmids from GP and typical STEC O157:H7 and SF STEC O157:H– suggest that a pO157-like plasmid might have been acquired by their common ancestor. Although more extensive studies are needed to obtain a complete understanding of these issues, only a limited number of SF STEC O157:H– genome sequences and no complete sequences of the Stx phages of this lineage are currently available. The available genome sequence information of GP STEC O157:H7 is also highly biased toward Japanese isolates.

In conclusion, we isolated a Stx2a-producing GP STEC O157:H7 strain that emerged from the *stx1a/stx2c*-positive GP STEC O157:H7 clone circulating in Japan; the virulence potential of this isolate is similar to that of typical STEC O157:H7. Researchers should pay more attention to this less commonly reported STEC O157 lineage, particularly the spread of this Stx2a-producing GP STEC O157:H7 clone and the emergence of additional *stx2a*-positive clones. Larger-scale genomic analyses including more GP STEC O157:H7 strains from various geographic regions and more SF STEC O157:H– strains are required to obtain a better understanding of the evolution and genomic diversity of GP O157:H7.

Technical Appendix Additional information about the various strains of *E. coli* used in this study of Shiga toxin–producing *E. coli* with the serotype O157:H7.
