# Correction: Vol. 24, No. 12

**DOI:** 10.3201/eid2502.C12502

**Published:** 2019-02

**Keywords:** errata, erratum, correction

In Figure 1 of the article Genomic Characterization of β-Glucuronidase–Positive *Escherichia coli* O157:H7 Producing Stx2a (Y. Ogura et al.), Shiga toxin–producing *Escherichia coli* O157 was mislabeled several times, and the term STEC was incompletely defined. The article has been corrected online (https://wwwnc.cdc.gov/eid/article/24/12/18-0404_article).

