# Supplementary material for: Genomic Characterization of β-Glucuronidase–Positive Escherichia coli O157:H7 Producing Stx2a
Source: Emerg Infect Dis. 2018 Dec;24(12):2219–27. doi: 10.3201/eid2412.180404 (PMC6256406; doi:10.3201/eid2412.180404)
Supplement: Technical Appendix — Additional information about the various strains of E. coli used in this study of Shiga toxin–producing E. coli with the serotype O157:H7. [file 18-0404-Techapp-s1.pdf]

Article DOI: https://doi.org/10.3201/eid2412.180404

Genomic Characterization of β-Glucuronidase–Positive *Escherichia coli* O157:H7 Producing Stx2a

Technical Appendix

Technical Appendix Table 1. *E. coli* strains used in this study

Strain	Serotype	GUD	stx Type	Isolation Year	Isolation Country	Host	Patient symptoms	Genome sequence status	Genome size or total scaffold length, bp	No. scaffolds	GenBank accession no.	Reference
Dec5d	O55:H7	+	negative	1965	Sri Lanka	Humans	Diarrhea	Draft	5,231,963	48	AIFT00000000	(*1*)
CB9615	O55:H7	+	negative	2003	Germany	Humans	Diarrhea	Complete	5,452,353	2	CP001846, CP001847	(*2*)
122262	O55:H7	+	*stx2a*	2014	UK	Humans	NA (Dorset outbreak isolate)	Draft	5,431,378	15	NZ_MINC00000000	(*3*)
493/89	O157:H-	+	*stx2a*	1989	Germany	Humans	HUS	Draft	5,359,584	351	AGTG00000000	Only sequence data available
LB473017	O157:H-	+	*stx2a*	2016	Germany	Humans	NA (HUS outbreak isolate)	Draft	5,444,136	211	ERR1989145	Only sequence data available
Sakai	O157:H7	–	*stx1a+stx2a*	1996	Japan	Humans	NA (Sakai outbreak isolate)	Complete	5,591,300	3	BA000007.3, AP018692	(*4*)
EDL933	O157:H7	–	*stx1a+stx2a*	1982	USA	Humans	NA (hamburger outbreak isolate)	Complete	5,639,399	2	CP008957, CP008958	(*5*)
EC4115	O157:H7	–	*stx2a+stx2c*	2006	USA	Humans	NA (Spinach outbreak isolate)	Complete	5,704,171	3	CP001164, CP001163	(*6*)
TW14359	O157:H7	–	*stx2a+stx2c*	2006	USA	Humans	NA (Spinach outbreak isolate)	Complete	5,622,737	2	CP001368, CP001369	(*7*)
SS52	O157:H7	–	*stx2a+stx2c*	2010	USA	Cattle	NA	Complete	5,583,430	2	CP010304, CP010305	(*8*)
155	O157:H7	–	*stx2a*	2012	UK	Humans	NA	Complete	5,604,428	2	CP018237, CP018238	(*9*)
180-PT57	O157:H7	–	*stx1a+stx2c*	2012	UK	Humans	Diarrhea	Complete	5,750,190	2	CP015832, CP015832	(*9*)
272	O157:H7	–	*stx2a*	2013	UK	Humans	NA	Complete	5,568,363	2	CP018239, CP018239	(*9*)
319	O157:H7	–	*stx1a*	2012	UK	Humans	NA	Complete	5,567,385	2	CP018241, CP018241	(*9*)

Strain	Serotype	GUD	stx Type	Isolation Year	Isolation Country	Host	Patient symptoms	Genome sequence status	Genome size or total scaffold length, bp	No. scaffolds	GenBank accession no.	Reference
350	O157:H7	–	*stx1a+stx2c*	2011	UK	Humans	NA	Complete	5,504,345	2	CP018243, CP018244	(*9*)
472	O157:H7	–	*stx1a+stx2c*	2012	UK	Humans	NA	Complete	5,608,872	2	CP018245, CP018246	(*9*)
7784	O157:H7	–	*stx2c*	2002	UK	Cattle	NA	Complete	5,462,063	2	CP018247, CP018248	(*9*)
9000	O157:H7	–	*stx2a+stx2c*	2002	UK	Cattle	NA	Complete	5,611,726	2	CP018252, CP018253	(*9*)
10671	O157:H7	–	*stx2c*	2002	UK	Cattle	NA	Complete	5,520,540	2	CP018250, CP018251	(*9*)
10.0869	O157:H7	+	*stx2c*	2010	USA	Cow	NA	Draft	5,267,107	257	AMTL01000000	Only sequence data available
G5101	O157:H7	+	*stx1a+stx2c**	1995	USA	Humans	Bloody diarrhea	Draft	5,061,556	217	NZ_AETX01000000	(*10*)
PV15-279	O157:H7	+	*stx1a+stx2a+ stx2c*	2015	Japan	Humans	Abdominal pain, vomiting, bloody diarrhea	Complete (this study)	5,692,637	2	AP018488, AP018489	This study
980938	O157:H7	+	*stx1a+stx2c*	1998	Japan	Humans	Asymptomatic carrier	Draft (this study)	5,326,010	239	BFBK01000000	This study
981447	O157:H7	+	*stx1a+stx2c*	1998	Japan	Humans	Abdominal pain, bloody diarrhea	Draft (this study)	5,353,000	222	BFBL01000000	This study
11278	O157:H7	+	*stx1a+stx2c*	1996	Japan	Humans	Symptomatic patient, details unknown	Draft (this study)	5,345,759	249	BFBC01000000	This study
11279	O157:H7	+	*stx1a+stx2c*	1996	Japan	Humans	Symptomatic patient, details unknown	Draft (this study)	5,350,253	254	BFBD01000000	This study
11280	O157:H7	+	*stx1a+stx2c*	1996	Japan	Humans	Asymptomatic carrier	Draft (this study)	5,351,942	193	BFBE01000000	This study
11281	O157:H7	+	*stx1a+stx2c*	1996	Japan	Humans	Symptomatic patient, details unknown	Draft (this study)	5,353,015	244	BFBF01000000	This study
13548	O157:H7	+	*stx1a+stx2c*	2013	Japan	Humans	Symptomatic patient, details unknown	Draft (this study)	5,389,595	221	BFBG01000000	This study
14152	O157:H7	+	*stx1a+stx2c*	1997	Japan	Humans	Unknown	Draft (this study)	5,392,172	228	BFBH01000000	This study
14153	O157:H7	+	*stx1a+stx2c*	1997	Japan	Humans	Asymptomatic carrier	Draft (this study)	5,358,401	232	BFBI01000000	This study
14156	O157:H7	+	*stx1a+stx2c*	1997	Japan	Humans	Symptomatic patient, details unkown	Draft (this study)	5,382,111	226	BFBJ01000000	This study
PV98-491	O157:H7	+	*stx1a+stx2c*	1998	Japan	Humans	Abdominal pain, bloody diarrhea	Draft (this study)	5,294,970	343	BFBN01000000	This study
PV98-623	O157:H7	+	*stx1a+stx2c*	1998	Japan	Humans	Fever, diarrhea	Draft (this study)	5,268,471	203	BFBO01000000	This study
PV00-061	O157:H7	+	*stx1a+stx2c*	2000	Japan	Humans	Abdominal pain, bloody diarrhea	Draft (this study)	5,555,028	318	BFBM01000000	This study

*GP O157:H7 strain G5101 has been reported to be *stx1/stx2*-positive, but *stx* genes was not detected by a blastn search in the genome sequence data of this strain obtained from public database. NA, not available.

Technical Appendix Table 2. Prophages and integrative elements in strains PV15-279 and Sakai and their integration sites and encoding virulence genes

Prophages								
PV15-279					Sakai			
ID	Length	Features	Virulence-related genes	Integration site	Virulence-related genes	Features	Length	ID
				tRNA(*thrW*)		Lambda-like phage	10586	Sp1
PV15p1	13316	P4-like phage				P4-like phage	12887	Sp2
PV15p2	21550	Lambda-like phage	*sfpA, nleB, nleC, nleH, nleD*	Intergenic (*ybhC-ybhB*)	*sfpA, nleB, nleC, nleH, nleD*	Lambda-like phage	38586	Sp3
PV15p3	8903	P4-like phage		Intergenic (*cspD-clpS*)				
PV15p4	46445	Lambda-like phage	*tccp2, espV*	tRNA(*serT*)	*pchA, tccp2, espV*	Lambda-like phage	49791	Sp4
				wrbA	*stx2a*	Lambda-like phage	62708	Sp5
PV15p5	17277	Lambda-like phage	*espX, espN, espO, espK*	*potB*	*espX, espN, espO, espK*	Lambda-like phage	48423	Sp6
PV15p6	10978	Untypeable	*pchE*	Intergenic (*roxA-phoQ*)	*pchE*	Untypeable	15461	Sp7
PV15p7	39433	Lambda-like phage		*icd*		Lambda-like phage	44388	Sp8
PV15p8	45257	Lambda-like phage	*ospG*	*ompW*	*paa, nleA, nleH, nleF, espO, nleG, espM, nleG*	Lambda-like phage	58082	Sp9
-				*ttcA*	*nleG, nleG, nleG*	Lambda-like phage	51155	Sp10
PV15p9	43822	Lambda-like phage	*paa, nleG, nleG, nleI*	*ydfJ*	*pchB, nleG, nleG, nleG*	Lambda-like phage	45778	Sp11
PV15p10	44098	Lambda-like phage	*stx2a, nleC*		*nleG, nleG, nleI*	Lambda-like phage	50939	Sp12
PV15p11	56336	Lambda-like phage	*nleG, espM, nleG, nleH, nleA, nleG*					
PV15p12	39692	Lambda-like phage	*pchB, nleG, nleG, nleG*					
PV15p13	14645	Untypeable	*pchE*	*tsqA*				
PV15p14	44075	Lambda-like phage	*stx2a, nleC*	*yecE*				
PV15p15	37482	P2-like phage		tRNA(*ileZ*)		P2-like phage	21120	Sp13
PV15p16	44636	Lambda-like phage	*pchC, tccP, espJ*	tRNA(*serU*)	*pchC, tccP, espJ*	Lambda-like phage	44028	Sp14
PV15p17	54660	Lambda-like phage	*stx1a, ospB, nleG*	*sbcB*				
PV15p18	58338	Lambda-like phage	*stx2c, espN, nleI*	*yehV*(*mlrA*)	*stx1a*	Lambda-like phage	47879	Sp15
PV15p19	9758	P22-like phage		*argW*		P22-like phage	8551	Sp16
PV15p20	11414	Untypeable		*eutA*				
PV15p21	49706	Lambda-like phage	*nleG, espW, nleG, espM*	tmRNA(*ssrA*)	*espW, nleG, espM*	lambda-like phage	24193	Sp17
				sorM		Mu-like phage	38759	Sp18
PV15p22	37935	Lambda-like phage	*pchA, nleG, nleG, nleG*	*dusA*				
Integrative elements								

Prophages									
	PV15-279					Sakai			
ID	Length	Features	Virulence-related genes	Integration site	Virulence-related genes	Features	Length	ID
PV15IE1	79594		Urease operon, tellurium resistance operon, iha adhesin gene, *pchD, aidA-I*	tRNA(*serX*)	Urease operon, tellurium resistance operon, iha adhesin gene, *pchD, aidA-I*		86248	SpLE1
				yeeX			13459	SpLE2
PV15IE2	20245		*efa1, espL, nleB, nleE*	*pheV*	*efa1, espL, nleB, nleE*		23451	SpLE3
PV15IE3	40984	LEE	T3SS machinery, *espG, espF, espB, tir, map, espH, espZ*	tRNA(*serC*)	T3SS machinery, *espG, espF, espB, tir, map, espH, espZ*	LEE	43450	SpLE4
PV15IE4	10216			*leuX*			10235	SpLE5
PV15IE5	37911						34148	SpLE6

Technical Appendix Table 3. Summmary of the comparison of insertion sequence (IS) elements between PV15-279 and Sakai

	Copy numbers in	
IS elements	PV15-279	Sakai
IS*1203* (IS*629*)	45	23
IS*Ec8*	19	12
IS*Ec1*	5	5
IS*30*	3	4
IS*100*	2	0
IS*1F*	2	1
IS*2*	2	1
IS*609*	2	2
IS*Ec31*	2	0
IS*1H*	1	1
IS*682*	1	1
IS*Ec13*	1	2
IS*Ec20*	1	0
IS*Ec22*	1	0
IS*Ec26*	1	1
IS*Ec47*	1	1
IS*Sfl3*	1	1
IS*Sd1*	0	4
IS*630*	0	2
IS*91*	0	2
IS*Ec23*	0	2
IS*Ec31*	0	2
IS*1414*	0	1
IS*911*	1	1
IS*Cro3*	0	1
IS*Ec2*	0	1
IS*Ec48*	0	1
IS*Ec62*	0	1
Total	91	73

Technical Appendix Table 4. Comparison of T3SS effectors encoded by prophages and integrative elements between PV15-279 and Sakai

	No. genes*	
Family	PV15-279	Sakai
EspF†	1	1
EspG†	1	1
EspH†	1	1
EspJ	1	1
EspK	1	1
EspL	1	1
EspM	2	2
EspN	2	1
EspO	1	2
EspV	1 (1)	1 (1)
EspW	1	1
EspX	1	1
EspZ†	1	1
Map†	1	1
NleA/EspI	1	1
NleB	3 (1)	3 (1)
NleC	3 (2)	1
NleD	1	1
NleE	1	1
NleF	0	1
NleG	16 (9)	14 (6)
NleH	1	2
TccP	2 (1)	2 (1)
OspB	1	0
OspG	1	0
Total	46 (14)	42 (9)

*Numbers of pseudogenes are indicated in parentheses.
†Encoded by the LEE element.

References

1. Hazen TH, Sahl JW, Redman JC, Morris CR, Daugherty SC, Chibucos MC, et al. Draft genome sequences of the diarrheagenic *Escherichia coli* collection. J Bacteriol. 2012;194:3026–7. http://dx.doi.org/10.1128/JB.00426-12

2. Zhou Z, Li X, Liu B, Beutin L, Xu J, Ren Y, et al. Derivation of *Escherichia coli* O157:H7 from its O55:H7 precursor. PLoS One. 2010;5:e8700. http://dx.doi.org/10.1371/journal.pone.0008700

3. Schutz K, Cowley LA, Shaaban S, Carroll A, McNamara E, Gally DL, et al. Evolutionary context of non-sorbitol-fermenting Shiga toxin-producing *Escherichia coli* O55:H7. Emerg Infect Dis. 2017;23:1966–73. http://dx.doi.org/10.3201/eid2312.170628

4. Hayashi T, Makino K, Ohnishi M, Kurokawa K, Ishii K, Yokoyama K, et al. Complete genome sequence of enterohemorrhagic *Escherichia coli* O157:H7 and genomic comparison with a laboratory strain K-12. DNA Res. 2001;8:11–22. http://dx.doi.org/10.1093/dnares/8.1.11

5. Latif H, Li HJ, Charusanti P, Palsson BO, Aziz RKA. A gapless, unambiguous genome squence of the enterohemorrhagic *Escherichia coli* O157:H7 strain EDL933. Genome Announc. 2014;2:e00821–14. http://dx.doi.org/10.1128/genomeA.00821-14

6. Eppinger M, Mammel MK, Leclerc JE, Ravel J, Cebula TA. Genomic anatomy of *Escherichia coli* O157:H7 outbreaks. Proc Natl Acad Sci U S A. 2011;108:20142–7. http://dx.doi.org/10.1073/pnas.1107176108

7. Kulasekara BR, Jacobs M, Zhou Y, Wu Z, Sims E, Saenphimmachak C, et al. Analysis of the genome of the *Escherichia coli* O157:H7 2006 spinach-associated outbreak isolate indicates candidate genes that may enhance virulence. Infect Immun. 2009;77:3713–21. http://dx.doi.org/10.1128/IAI.00198-09

8. Katani R, Cote R, Raygoza Garay JA, Li L, Arthur TM, DebRoy C, et al. Complete genome sequence of SS52, a strain of *Escherichia coli* O157:H7 recovered from supershedder cattle. Genome Announc. 2015;3:e01569–14. http://dx.doi.org/10.1128/genomeA.01569-14

9. Shaaban S, Cowley LA, McAteer SP, Jenkins C, Dallman TJ, Bono JL, et al. Evolution of a zoonotic pathogen: investigating prophage diversity in enterohaemorrhagic *Escherichia coli* O157 by long-read sequencing. Microb Genom. 2016;2:e000096.

10. Rump LV, Strain EA, Cao G, Allard MW, Fischer M, Brown EW, et al. Draft genome sequences of six *Escherichia coli* isolates from the stepwise model of emergence of *Escherichia coli* O157:H7. J Bacteriol. 2011;193:2058–9. http://dx.doi.org/10.1128/JB.00118-11

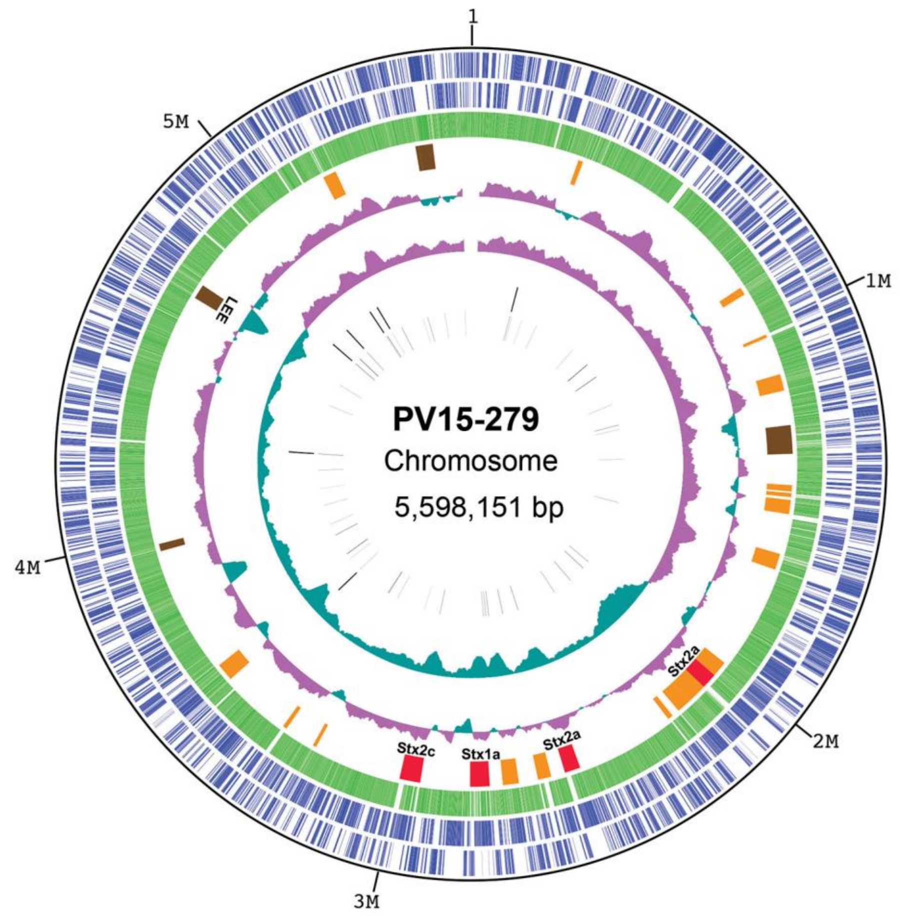

Technical Appendix Figure 1. A circular map of the PV15-279 chromosome is shown. From the outside in: 1st circle, nucleotide sequence positions (in Mb); 2nd and 3rd circles, coding sequences (CDSs) transcribed clockwise and counterclockwise, respectively; 4th circle, CDSs conserved in Sakai (≥90% identity and ≥50% coverage); 5th circle, locations of prophages (PPs) and integrative elements (IEs) (red: Stx PPs, orange: other PPs, brown: IEs); 6th circle, GC skew; 7th circle, GC content; 8th circle, rRNA; 9th circle, tRNA.

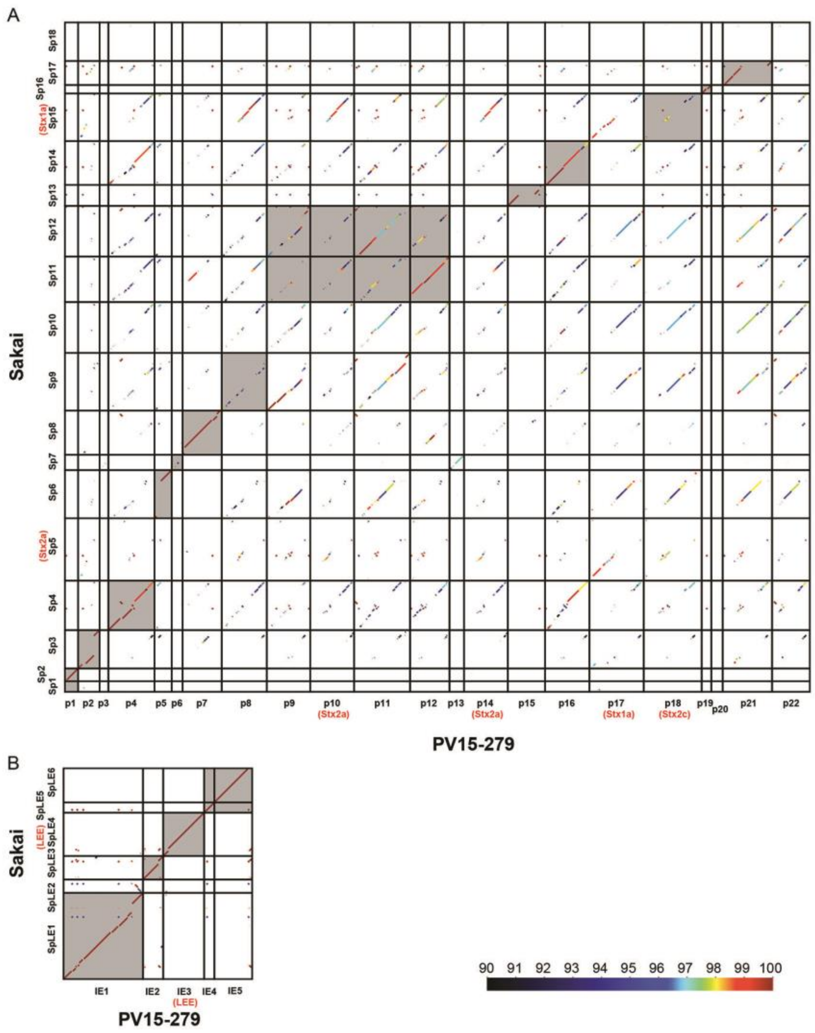

Technical Appendix Figure 2. Sequence comparisons of all the phages and integrative elements of PV15-279 and Sakai. The dot plot matrices of all the phages (A) and integrative elements (B) of PV15-279 and Sakai are shown. Sequence identities are indicated by different colors. Phages and elements integrated at the same integration sites are highlighted with gray shading.

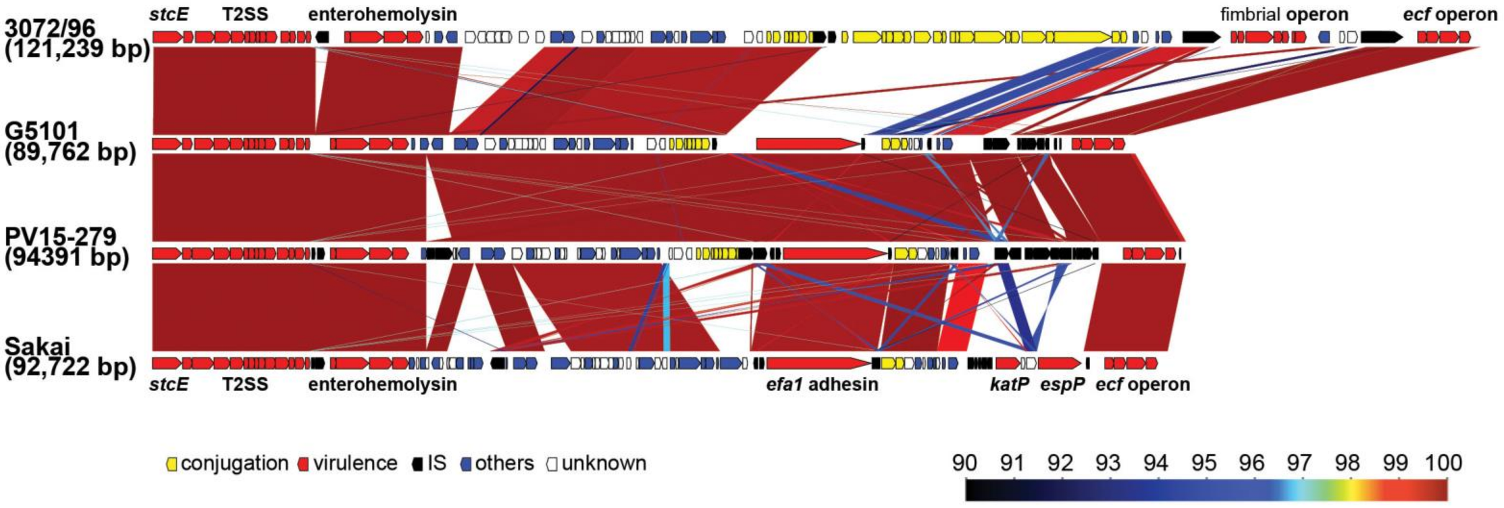

Technical Appendix Figure 3. Structural comparison of the virulence plasmids of typical O157:H7, SF O157:H–, and GP O157:H7. The structures of the virulence plasmids of the typical O157:H7 strain Sakai (GenBank accession no. AB011548), the GP O157:H7 strains PV15-279 (GenBank accession no. AP018489) and G5101 (GenBank accession no. AETX01000217) and the SF O157:H– strain 3072/96 (GenBank accession no. AF401292) are shown. Homologous regions are indicated by shading, and sequence identities are indicated by different colors.
